# Takotsubo Cardiomyopathy in the Emergency Department: A FOCUS Heart Breaker

**DOI:** 10.5811/cpcem.2018.2.37291

**Published:** 2018-04-05

**Authors:** Kristin Meigh, Madison Caja, Melinda Sharon, Allison Tadros, Shane Dragan, David Henkel, Joseph Minardi

**Affiliations:** * West Virginia University School of Medicine, Morgantown, West Virginia; † West Virginia University, Department of Emergency Medicine, Morgantown, West Virginia; ‡ West Virginia University, Department of Medical Education, Morgantown, West Virginia

## Abstract

Takotsubo cardiomyopathy (TCM) is an important condition for the emergency physician to consider in patients with cardiovascular symptoms. A 70-year-old woman presented with chest pain and nausea following emotional trauma. She had an elevated troponin and a normal electrocardiogram with no history of previous cardiac disease. Point-of-care focused cardiac ultrasound (FOCUS) showed reduced left ventricular systolic function with mid to apical hypokinesis. Cardiac catheterization revealed clean coronary arteries and confirmed the suspected diagnosis of TCM. Few reports emphasize the importance of FOCUS in the diagnosis and management of TCM in the emergency department. We detail FOCUS findings that assisted with diagnosis of TCM and describe how this quick, noninvasive imaging modality can be used to assess and manage emergent conditions.

## INTRODUCTION

Takotsubo cardiomyopathy (TCM) is an important differential consideration for emergency patients with cardiovascular symptoms. Also known as broken heart syndrome or stress-induced cardiomyopathy, this condition typically occurs in elderly women following a stressful psychological or physical event.^1^ Patients typically present with symptoms of an acute coronary syndrome or new-onset heart failure, and may show accompanying changes such as elevated troponin level and ST-segment elevation on the 12-lead electrocardiogram (ECG).

Point-of-care focused cardiac ultrasound (FOCUS) is a critical diagnostic tool that should be implemented by emergency physicians (EP) in patients with chest pain and suspected TCM. FOCUS has been previously reported to help diagnose other conditions on the differential such as pulmonary embolism or pericardial tamponade.^2^,^3^ Other reports that have described the use of FOCUS by EPs for TCM have been less descriptive or have focused on atypical presentations of TCM.^4^,^5^ In comparison, our case involves a classic presentation of the condition associated with a high index of suspicion in the emergency department (ED). Our aim was to describe the role of FOCUS in efficiently managing these patients. Using FOCUS to help diagnose TCM and distinguish it from other cardiac emergencies will enable EPs to better recognize and treat this important condition.

## CASE REPORT

A 70-year-old woman presented to the ED with chest pain and nausea. The symptoms started after receiving news about a family member’s critical illness. The chest pain was described as heavy with radiation to her right upper extremity and was associated with dyspnea. No other symptoms were associated with the chest pain. She had no history of previous cardiac disease. She was a smoker and her past medical history was significant for hyperlipidemia.

**Image 1:**
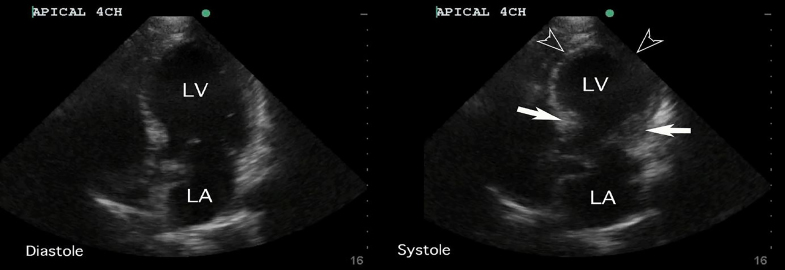
This apical 4-chamber view of the heart performed by the emergency physician demonstrates findings of apical ballooning, with systolic mid to apical hypokinesis of the left ventricle (LV). The basal segments near the atrioventricular septum contract appropriately in systole (solid arrows), but the mid to apical segments of the left ventricle show minimal contraction and demonstrate ballooning when systole is compared to diastole (outlined arrowheads). These findings match the description of the classic apical variant of takotsubo cardiomyopathy, which is said to resemble an octopus pot. *LA,* left atrium.

**Image 2:**
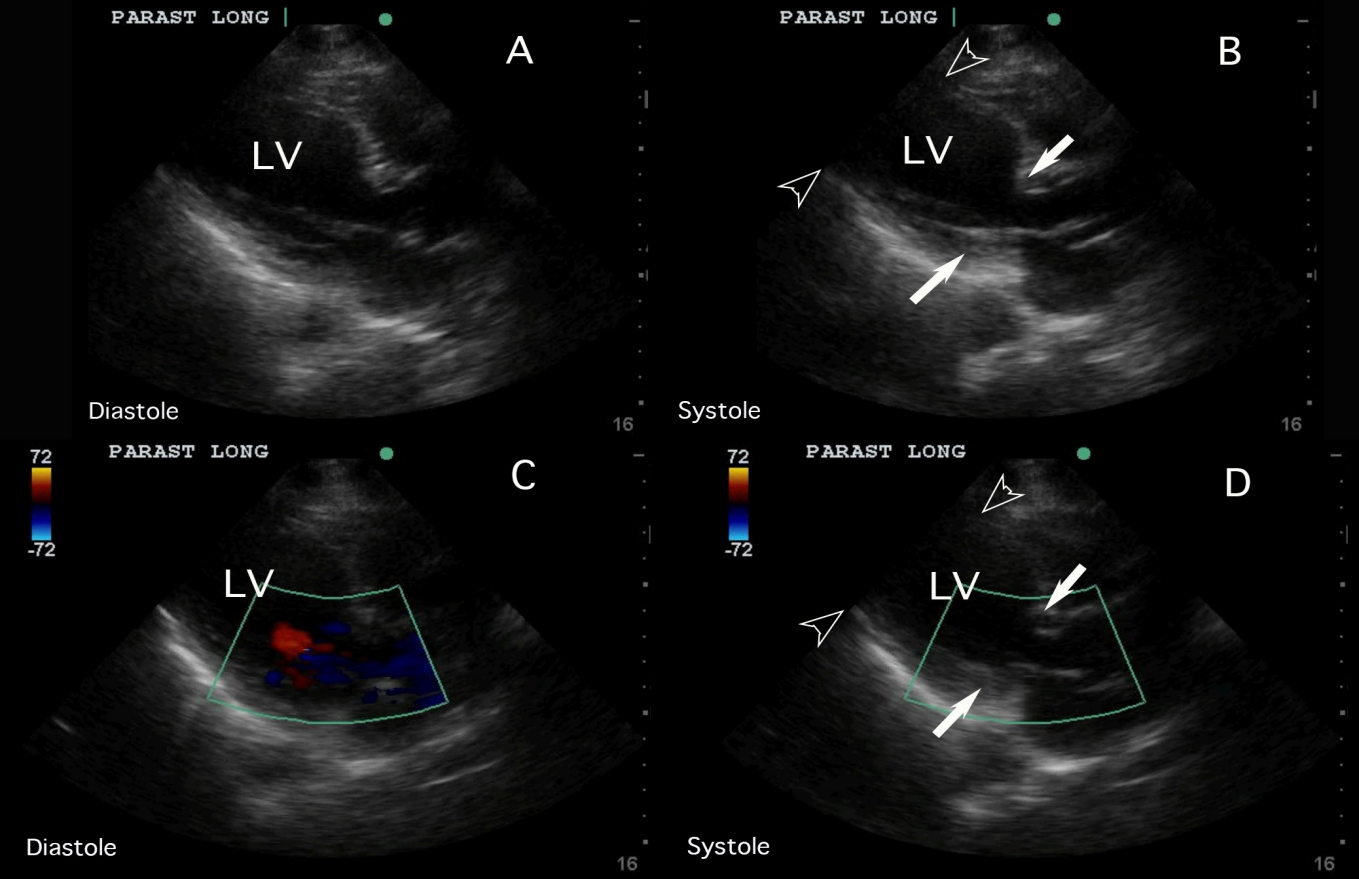
These parasternal long-axis views, both with (panels C and D) and without (panels A and B) color, demonstrate the systolic apical ballooning of the left ventricle (LV) with preserved contraction of the basal segments. The apical portions of the interventricular septum and free wall of the left ventricle do not show significant movement when systole (panels B and D) is compared to diastole (panels A and C), indicating impaired contraction of the apical segment (outlined arrowheads). The left ventricular basal segments, however, move closer together in systole (panels B and D) to show preserved contraction of this portion of the heart (solid arrows).

Her vital signs were unremarkable, with heart rate of 84 beats per minute, respiratory rate of 18 breaths per minute, blood pressure of 124/84 mmHg, and temperature of 36.7° Celsius. Her physical examination was also unremarkable. Initial ECG demonstrated normal sinus rhythm. Chest radiograph showed hyperexpanded lungs with chronic obstructive changes but revealed no acute process. FOCUS performed by a general EP without fellowship training or special interest in ultrasound revealed severely reduced left ventricular systolic function with mid to apical hypokinesis and preservation of basal segments (Video). The apical 4-chamber and parasternal long axis FOCUS findings can be observed in Images 1 and 2, respectively.

Takotsubo cardiomyopathy was strongly suspected based on these findings and the patient’s presentation. She was treated with aspirin and typical measures for acute coronary syndrome and admitted to the cardiology service. Her troponin returned at 595 ng/L and a subsequent ECG showed lateral T-wave inversions. There were no significant changes in vital signs from time of presentation. She underwent a comprehensive echocardiogram that confirmed the FOCUS findings. Because her troponins continued to rise, she underwent a cardiac catheterization the following day, which revealed clean coronary arteries and supported the diagnosis of TCM. Other than the development of atrial fibrillation, her hospital course was unremarkable and she was discharged a few days later. Follow-up echocardiogram seven weeks later demonstrated normal left ventricular function. She was no longer in atrial fibrillation, and the patient reported her symptoms had resolved.

## DISCUSSION

TCM represents approximately 1.2% of troponin-positive acute coronary syndromes.^6^ The term *takotsubo* comes from the Japanese word for “octopus pot,” which the appearance of the affected patient’s heart resembles, typically showing hypokinesis or ballooning of the apical segments and hyperkinesis of the basal segments.^7^ This description matches the most common form of TCM, known as apical TCM. According to the International Takotsubo Registry (ITR), a rare variant of TCM known as basal or reverse takotsubo, occurs in approximately 2.2% of TCM patients.^1^ In this form, the opposite presentation is seen, with hypokinesis of the basal heart and hyperkinesis of the apical segments.^1^ Two other variants, focal and midventricular TCM, have also been described.^1^

**CPC-EM Capsule**

What do we already know about this clinical entity?

Takotsubo cardiomyopathy is a type of stress-induced left ventricular dysfunction that is an important consideration in the differential diagnosis for chest pain.

What makes this presentation of disease reportable?

Unlike previous reports, this is a classic clinical presentation with well demonstrated focused cardiac ultrasound (FOCUS) findings that directed immediate care decisions.

What is the major learning point?

Chest pain after emotional upset with apical ballooning on FOCUS suggests Takotsubo cardiomyopathy, and should give pause to thrombolytics and prompt consideration of alternate care plans.

How might this improve emergency medicine practice?

Recognizing classic findings of Takotsubo cardiomyopathy may alter differentials and prompt alternate care plans, specifically when immediate cardiac catheterization is not available.

FOCUS assists in identifying the classic findings that may suggest TCM. FOCUS has been proven to be beneficial in diagnosing a myriad of other cardiorespiratory conditions such as atypical pericardial tamponade, pulmonary embolism, endocarditis, intra-cardiac masses, atypical acute coronary syndromes, and more.^2^,^3^,^8^,^9^ Despite the importance of FOCUS in diagnosing other acute cardiac conditions, few reports exist that detail its value in visualizing findings of TCM and subsequently narrowing the differential diagnosis. A recent case study described the use of FOCUS in an atypical presentation of TCM.^5^

The diagnosis was not readily suspected with the patient’s history, as she was a pre-menopausal woman with no recognizable triggering event. An EP performed FOCUS and noted apical ballooning and reduced LVEF. A comprehensive echocardiogram was subsequently performed by cardiology to confirm the findings, and prompt cardiac catheterization confirmed a final diagnosis of TCM. An additional case compared cardiac ultrasonography findings from the ED to cardiac catheterization images to demonstrate apical ballooning in a post-menopausal woman with a significant cardiac history.^4^ In comparison to the previous reports in the literature, our case study focused on a more classic presentation of this disease in the ED where there was a high index of suspicion; it highlights how FOCUS can be used to expedite this diagnosis.

The Mayo Clinic Criteria are typically used for the diagnosis of TCM.^10^ All four criteria must be present to correctly diagnose the condition, and are as follows:

- Transient LV systolic dysfunction extending beyond a single coronary artery territory
- Absence of obstructive coronary artery disease on cardiac catheterization
- New ECG abnormalities (ST-elevation or T-wave inversion) or troponin elevation
- Absence of myocarditis or pheochromocytoma.

These criteria are designed in part to assist physicians in distinguishing TCM from other acute cardiac conditions. Coronary angiography (CA) is considered the gold standard for differentiating TCM from acute myocardial infarction (AMI) by ruling out obstructive disease of the coronary arteries. However, FOCUS may be preferred, both before and after CA, due to its increasing availability, rapidity, and effectiveness.^11^ FOCUS allows for the rapid detection of both apical and alternative forms of TCM, since the site of systolic dysfunction in the left ventricular wall can readily be visualized with this imaging method.

After recognition of wall motion abnormalities on FOCUS, coronary computed tomography angiography (CCTA) may be considered instead of CA to rule out coronary artery disease (CAD), due to the fact that CCTA is noninvasive and has a high negative predictive value for CAD.^11^ Pairing CCTA with FOCUS could allow for a relatively noninvasive diagnosis of TCM in future patients compared to current standards. While ECG and troponin changes can also assist in this diagnosis, it is difficult to distinguish between TCM and AMI based on these parameters alone; therefore, FOCUS can provide additional useful data to help distinguish these important acute conditions.^11^ This is especially critical when fibrinolytic therapy is being considered for an AMI, as inappropriate administration of this treatment could lead to unwanted adverse affects.^12^

Understanding of the epidemiology and risk factors associated with TCM can also assist the EP in evaluating these patients. TCM classically presents in post-menopausal females exposed to a physically or emotionally stressful event. However, TCM may also occur in males with a physical trigger, such as an infection, or with no identifiable trigger.^1^,^13^ While classic apical TCM is more commonly associated with heart failure symptoms like dyspnea and pulmonary edema, reverse TCM typically occurs more frequently in younger patients.^14^,^15^ Echocardiogram parameters such as left ventricular ejection fraction (LVEF) at the time of diagnosis and follow-up have been found to be similar between reverse TCM and other variants.^14^

Compared with an acute coronary syndrome patient, patients with TCM more commonly demonstrated a lower LVEF and a higher incidence of neurological and psychiatric conditions.^1^ It is important to be aware of the differences between variants and to differentiate TCM from AMI, so that the diagnosis is not missed. Care must be taken and all clinical data carefully considered because echocardiographic and clinical findings of TCM may closely mimick those of acute coronary syndrome. It should be emphasized that coronary imaging is required for definitive exclusion of acute coronary syndrome and diagnosis of TCM.

The treatment for TCM patients is debated throughout the literature. Though beta-blockers are typically used for TCM patients due to the widely accepted role of catecholamines in the condition, the International Takotsubo Registry reports that ACE-inhibitors or angiotensin receptor blockers are more associated with improved survival of TCM patients.^1^ In addition, the prognosis also varies. Compared to ACS patients, TCM patients were found to have just as many if not more complications such as cardiogenic shock, cardiopulmonary arrest, and even death.^1^ Rapid recognition of TCM with FOCUS will help direct appropriate diagnostic workup and may prevent administration of potentially harmful therapy such as fibrinolytic therapy for an AMI. It will also assist in narrowing the differential for other cardiovascular conditions such as pulmonary embolism and pericardial effusions.^2^,^3^ Maintaining a high index of suspicion for TCM and using FOCUS to recognize wall motion abnormalities may help differentiate this condition from AMI, and will assist EPs in better management of these patients. Future studies could evaluate the utility of echocardiography paired with CCTA or other noninvasive pathways to make this diagnosis in select patients.

## CONCLUSION

Takotsubo cardiomyopathy is an important condition for the EP to consider in patients with cardiovascular symptoms. Recognizing the clinical presentation and FOCUS findings may help EPs make a more accurate and timely diagnosis as well as direct immediate management decisions for this important ACS mimic. While this diagnosis may be suspected based on initial ED evaluation including FOCUS, acute ischemia can only be excluded by coronary artery evaluation. Current practice requires invasive coronary angiography rule-out ACS, but a strategy that involves the use of FOCUS combined with CCTA in consultation with an interventional cardiologist may allow a completely noninvasive evaluation and confirmation of diagnosis in select patients.

Documented patient informed consent and/or Institutional Review Board approval has been obtained and filed for publication of this case report.

## Supplementary Information

Video Echocardiogram parasternal long-axis and apical 4-chamber views, both with and without color, displaying the systolic basal pinch points and apical ballooning. Parasternal short-axis and subxiphoid views show reduced left ventricular systolic function.
